# Cardiac Myosin-Binding Protein C—From Bench to Improved Diagnosis of Acute Myocardial Infarction

**DOI:** 10.1007/s10557-018-6845-3

**Published:** 2019-01-08

**Authors:** Thomas E. Kaier, Bashir Alaour, Michael Marber

**Affiliations:** King’s College London BHF Centre, The Rayne Institute, St Thomas’ Hospital, 4th Floor Lambeth Wing, Westminster Bridge Road, London, SE1 7EH UK

**Keywords:** cMyC, Cardiac myosin-binding protein C, Cardiac troponin, Chest pain, Triage, Biomarkers, Acute myocardial infarction, AMI

## Abstract

Chest pain is responsible for 6–10% of all presentations to acute healthcare providers. Triage is inherently difficult and heavily reliant on the quantification of cardiac Troponin (cTn), as a minority of patients with an ultimate diagnosis of acute myocardial infarction (AMI) present with clear diagnostic features such as ST-elevation on the electrocardiogram. Owing to slow release and disappearance of cTn, many patients require repeat blood testing or present with stable but elevated concentrations of the best available biomarker and are thus caught at the interplay of sensitivity and specificity.

We identified cardiac myosin-binding protein C (cMyC) in coronary venous effluent and developed a high-sensitivity assay by producing an array of monoclonal antibodies and choosing an ideal pair based on affinity and epitope maps. Compared to high-sensitivity cardiac Troponin (hs-cTn), we demonstrated that cMyC appears earlier and rises faster following myocardial necrosis. In this review, we discuss discovery and structure of cMyC, as well as the migration from a comparably insensitive to a high-sensitivity assay facilitating first clinical studies. This assay was subsequently used to describe relative abundance of the protein, compare sensitivity to two high-sensitivity cTn assays and test diagnostic performance in over 1900 patients presenting with chest pain and suspected AMI. A standout feature was cMyC’s ability to more effectively triage patients. This distinction is likely related to the documented greater abundance and more rapid release profile, which could significantly improve the early triage of patients with suspected AMI.

## Background

Despite it being a frequent occurrence in emergency departments (ED) around the world, chest pain triage remains a challenge for patients and physicians alike. Responsible for 6–10% of all presentations to acute healthcare providers [1–4], the presenting complaint of chest pain results in a high rate of admissions (1:3, according to data from the UK [5]), but a paradoxically low probability (10%) of a final diagnosis of acute myocardial infarction (AMI) [6]. The inability to make a rapid and accurate diagnosis not only causes financial but also medical, psychological and social burden to the affected patient and the healthcare system. Only 32% of patients with an ultimate diagnosis of AMI have diagnostic ECG changes of ST-elevation or depression that facilitate immediate triage [7, 8], in many cases—and healthcare environments—directly to heart attack centres. The remaining two-thirds of all patients eventually diagnosed with an acute coronary syndrome (ACS) present with non-ST elevation myocardial infarction (NSTEMI) [6]. Consequently, triage has become reliant on quantifying the biomarker cardiac Troponin (cTn). This is enshrined in the Universal Definition of Myocardial Infarction [9] (now in its fourth iteration [10]) by mandating the detection of a cardiac biomarker rise and/or fall for the diagnosis of AMI. Historically, patients tested with contemporary cTn assays had to wait for at least 12 h for a reliable diagnosis—on the basis that the cardiac-restrict troponin isoforms (cTnI and cTnT) are released slowly after myocardial injury and reach their respective peak concentration after 18 h [11, 12]. To facilitate earlier rule-in and rule-out of AMI, the cTn assay vendors then increased the analytic performance—to achieve high-sensitivity (hs), or, in simple terms, quantify cTn in the majority of patients. The ability to detect ever-lower concentrations of cTn enables direct rule-out of AMI in a specific subgroup—i.e. in patients with symptoms for more than 3 h, a normal ECG and an undetectable cTn level. While the European guidelines recommend the ‘measurement of a biomarker of cardiomyocyte injury, preferably high-sensitivity cardiac Troponin’ in all patients with suspected NSTEMI [13], the clinical implications of using hs-cTn assays include a 2-fold increase of detection of type 2 AMI, ~ 20% relative increase in detection of type 1 AMI and—all according to the ESC’s 2015 guideline—‘elevations up to 3-fold the upper reference limit (URL)… may be associated with a broad spectrum of conditions’. The very definition of a hs-cTn assay—according to the International Federation of Clinical Chemistry and Laboratory Medicine Task Force on Clinical Applications of cardiac Bio-Markers (IFCC TF-CB)—includes (1) a CV ≤ 10% at the 99th centile value and (2) the ability to measure at least 50% of healthy individuals with concentrations above the assay’s limit of detection (LoD) [14, 15]. Acknowledging the underlying biology, the ESC hence advocates the use of its 0/1 h rule-out/rule-in algorithm only in patients presenting > 3 h after chest pain onset. Several publications have recently reported on the variable effectiveness of the ESC algorithm in clinical practice—many patients have to undergo a second blood draw for a more refined triage, and only 20–30% of patients benefit from immediate rule-out/-in using the cut-offs published [16–19]. Taken together, technological advances result in many more patients being tested ‘Troponin-positive’, without necessarily being ‘AMI-positive’—while impressive with respect to assay development, cTn was inherently unsuited for early diagnosis of acute myocardial injury and this has not been mitigated by moving detection limits to ever-lower levels.

## Can We Do Better?

From the synopsis above, it is clear that new biomarkers are needed but the only way they can usurp cTn is if they possess equivalent cardiac selectivity but (1) rise more rapidly after acute myocardial injury (advances sensitivity) and/or (2) have a lower ‘background’ concentration in those with vascular risk factors or underlying chronic heart disease (advances specificity).

The ideal biomarker for early diagnosis of an acute coronary syndrome would have a release profile that is temporally analogous to cytosolic proteins (such as creatine kinase, fatty-acid binding protein and myoglobin) but possesses the cardiac-restricted expression of cardiac Troponins. Our group has identified cardiac myosin-binding protein C (cMyBP-C, cMyC; UniProtKB—Q14896) as a candidate marker [20], a cardiac sarcomeric protein which is at least twice as abundant in the heart as cTnI or cTnT [21]. We have shown it is released into the serum after myocardial infarction in the mouse [20] and in patients [22], findings which have been confirmed by others [23].

## Discovery and First Description of Cardiac Myosin-Binding Protein C (cMyC)

Originally described as the C-protein by Offer et al. in 1973 [24], its discovery relied on the characterisation of ‘impurities’ detected alongside myosin in sodium dodecyl sulphate (SDS) polyacrylamide gel electrophoresis. The resulting bands were labelled alphabetically, the third heaviest being correctly identified at the band corresponding to a molecular weight of 140 kDa. Offer et al. hypothesised that the protein’s main function might be that of a core protein, it might control or modify the movement of cross-bridges, or ‘serve a purely mechanical function’ [24]—preserving integrity and stabilising the filaments.

## Structure of cMyC

**Fig. 1:**
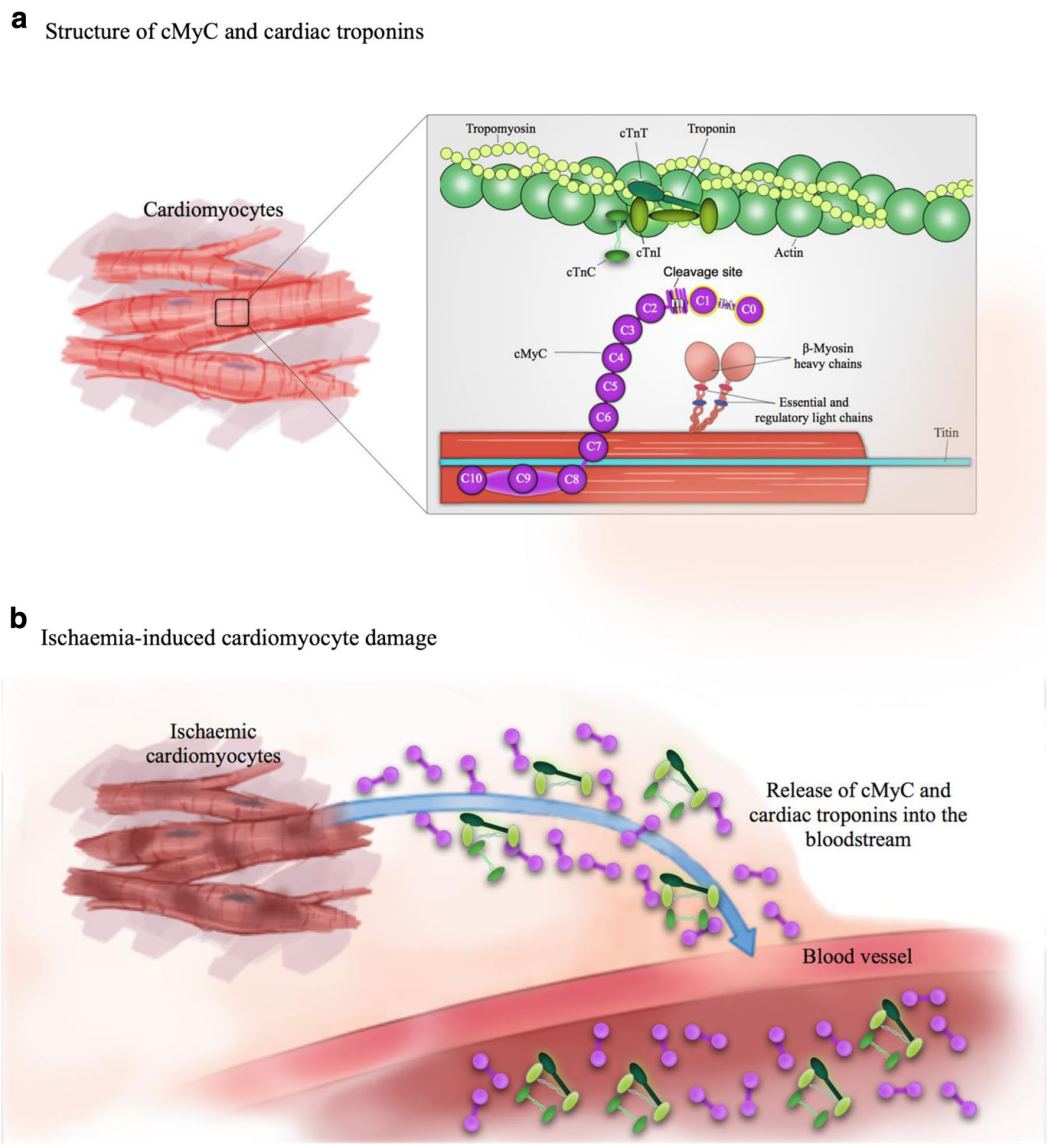
Structure of cMyC and relationship with the cTn complex; adapted from Kaier et al. [27]

Three isoforms of MyBP-C exist in adult human muscle—fast and slow skeletal (encoded by MYBPC1 and MYBPC2 genes on chromosomes 12q23.3 and 19q33.3, respectively), and a cardiac isoform (cMyBP-C, gene MYBPC3 on chromosome 11p11.2) [25, 26]. Uniquely, the cardiac isoform contains an additional immunoglobulin-like domain at the N terminus (C0), phosphorylation sites in between domains C1 and C2 (M motif) and a 28 amino acid insertion in the C5 domain. The whole protein consists of 12 domains, of which there are 8 immunoglobulin (IgC2)-like, 3 fibronectin (FN3) domains, plus the M domain mentioned above (Fig. 1).

The four phosphorylation sites, designated A–D (residue code for A, RRTS [272–275]; B, RRIS [281–284]; C, KRDS [301–304]; D, KKST [259–263]) by Gautel et al. [26], are, among others, phosphorylated by protein kinase A (PKA; for sites A, B and C), protein kinase C (PKC) and calmodulin kinase (CAMK; for site B) [28–30]. It appears that folding of the protein prohibits access to site D [31].

In 2008, Luther et al., using electron microscopy, imaged nine bands of cMyBP-C crossing the thick and thin filaments in perpendicular orientation in the C-zones of the A-band [32]. Still, to date, the exact arrangement in the sarcomere remains unclear, and two models are being tested: (1) a trimeric collar model, where three cMyBP-C molecules form a collar around the thick filament core [33]; and (2) a rod model where cMyBP-C interacts with its C-terminal domains along the thick filament axis, with the N-terminal domains extending towards the thin filament [34].

## Function of cMyC

The uncertainty regarding the exact structural arrangement is further reflected in an incomplete understanding of the interaction between cMyBP-C and thick and thin filaments. Better understood are the effects of cMyBP-C phosphorylation, which is necessary for normal myocardial function and appears to protect from ischaemic injury [35, 36]. These effects are predominantly mediated by phosphorylation at Ser-273, Ser-282 and Ser-302 sites, which diminish after ischaemia/reperfusion injury, or in the context of heart failure and hypertrophy [36], atrial fibrillation [37] or in cardiomyopathies [38]. More specifically, mouse models have shown that loss of phosphorylation (through phospho-ablation by residue substitution) is sufficient to cause hypertrophy and cardiac dysfunction [36, 39]. In the context of normal function, phosphorylation itself drives actin–myosin interaction and subsequently increases cross-bridge cycling rate—which in turn enhances cardiac contractility [40–43].

## Hypertrophic Cardiomyopathy

Gene defects affecting cMyBP-C have been extensively studied since the first description of two mutations causing hypertrophic cardiomyopathy (HCM) in separate kindreds 1995 [44, 45]. Better understood are the pathological consequences of gene defects affecting cMyBP-C. HCM affects about 0.25–1% of the population worldwide [46–48], and mutations in cMyBP-C are responsible for about one-third of symptomatic cases [49]. There are more than 350 unique mutations affecting cMyBP-C described to date [50] (for an up-to-date list, see uniprot.org [51]), > 60% of mutations are C′-truncations—and are, intriguingly, rarely detected by western blot of myocardium from affected HCM patients [49] (in the mouse model, a homozygous C′-truncation results in cMyBP-C null mouse hearts—equivalent to a homozygous knockout). This observation is attributed to cell surveillance mechanisms that protect affected cells from the adverse effects of the truncated proteins [52]. Thus, the phenotype of HCM is felt to be due to haploinsufficiency (a subtle reduction of the amount of cMyBP-C protein expressed since the healthy allele cannot fully compensate for the lack of protein expressed from the diseased allele) [53]. This reduces the overall amount of cMyBP-C expressed, but means the protein that is expressed is normal and unaffected [54]. The other pathogenic variants of cMyBP-C are missense mutations, resulting in single amino acid substitutions, with a range of associated phenotypes (from benign to severe). While they occur throughout the cMyBP-C protein [49], the domain linking C0 and C1 (enriched with proline and alanine residues; PA) seems to be exempt. More importantly, with a view to immunoassay development, most missense mutations affect the C-terminal domains beyond C3. It also remains unclear whether, and how, individual missense mutations cause disease. Proposed effects are alteration of domain folding, direct impairment of the cMyBP-C function or, again, haploinsufficiency. [49]

## Development of the In-House cMyC Immunoassay

**Fig. 2:**
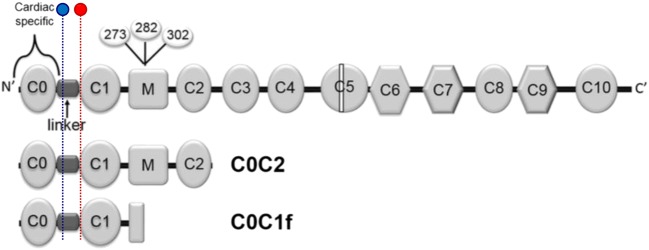
Structure of full-length cMyBP-C. Phosphorylation sites involved in the regulation of myocardial contractility—Ser-273, Ser-282 and Ser-302—highlighted in the M-domain (where calpain-dependent cleavage occurs) [55], and commonly detected N-terminal fragments, C0C2 and C0C1f. Binding sites for antibodies 1A4 (blue) and 3H8 (red) are highlighted. Reproduced and adapted from Lipps et al. [56]

**Fig. 3:**
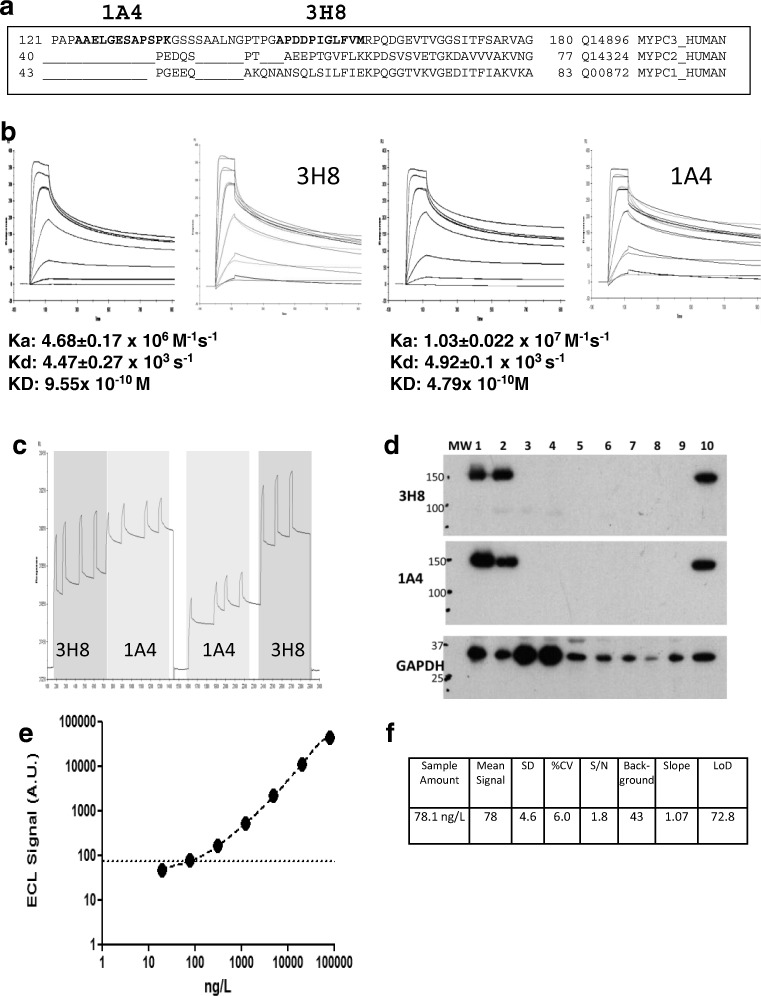
The development of a quantitative immunoassay for human cMyC in serum. **a** Sequence alignment of cMyC with skeletal myosin binding protein C isoforms. The sequence recognised by monoclonal anti-cMyC antibodies 1A4 and 3H8 are shown in bold. The antibodies bind to cardiac-restricted sequences with organ specificity further verified by immunoblots (see **d**). **b** SPR kinetic sensorgrams demonstrating the kinetic parameters of clone 3H8 (left) and 1A4 (right). These antibodies were selected from over 50 hybridomas, and both antibodies are of high affinity. **c** Epitope competition sensorgram of 1A4 and 3H8 binding to the C0C2 region of cMyC conjugated to a CM5 biosensor chip. Although antibodies recognise near adjacent epitopes, there is no appreciable interference between them. Near adjacency is needed since cMyC is fragmented in the circulation raising the possibility of separation of capture and detection epitopes if they were widely spaced. **d** Immunoblot of rat and human tissue demonstrating specificity of 3H8 and 1A4 monoclonal antibodies. GAPDH was used as a loading control. Samples 1–9 are various rat tissue (1 = ventricle, 2 = atria, 3 = rectus abdominus, 4 = soleus, 5 = spleen, 6 = kidney, 7 = aorta, 8 = liver, 9 = brain) and 10 is human ventricle. **e** Representative C0C2 standard curve from cMyC ECL assay indicating the limit of detection (dashed line). This in-house assay on a MesoScale Discovery enhanced chemiluminescent detection platform was used to measure cMyC appearance and disappearance in Figs. 2 and 3 below. Panel (**f**) demonstrates the performance characteristics of the assay, with a LoD of approximately 80 ng/L. Figures and legend reproduced from Baker et al. [22]

Over the past years, our group established and improved the analytic performance of the assay for cMyC—initially as an in-house assay which is described in detail by Baker et al. [22] The best-performing antibodies (clone 3H8 and clone 1A4) were selected for the creation of a ‘sandwich’ electrochemiluminescence assay (MesoScale Discovery (MSD), Sector imager 2400). The standard curve was used to quantify and express cMyC concentrations as nanograms per litre. This achieved an LLoQ of 80 ng/L (Figs. 2 and 3).

## In Vivo Models of Myocardial Infarction

**Fig. 4:**
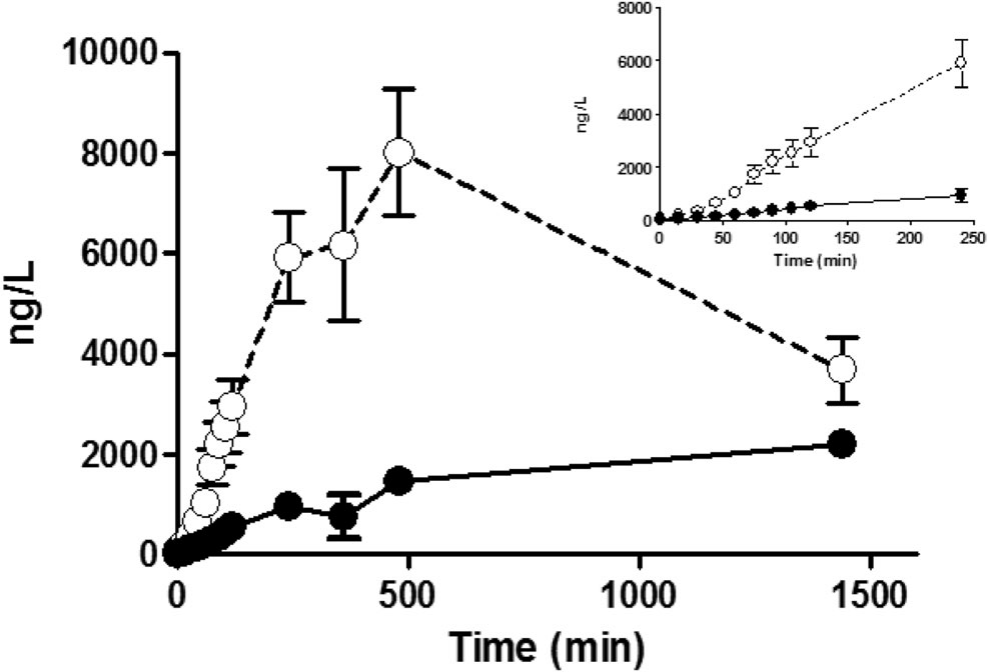
The accumulation of cMyC vs. cTnT after myocardial injury caused by intracoronary ethanol. Venous blood was collected frequently over the first 2 h, and up to 24 h, after therapeutic alcohol septal ablation for hypertrophic cardiomyopathy (TASH) using ethanol infused selectively into a septal perforating branch coronary artery. Summary data of absolute quantification of cMyC (open symbols) vs. cTnT (closed symbols) over time following TASH (*n* = 20). Inset figure is a zoom of the first 240 min. Over this time interval, cMyC accumulates in the serum approximately six times faster than cTnT (slope 25.8 ± 1.9 vs. 4.0 ± 0.4 ng/L/min, *p* < 0.0001). Figure reproduced from Baker et al. [22]

**Fig. 5:**
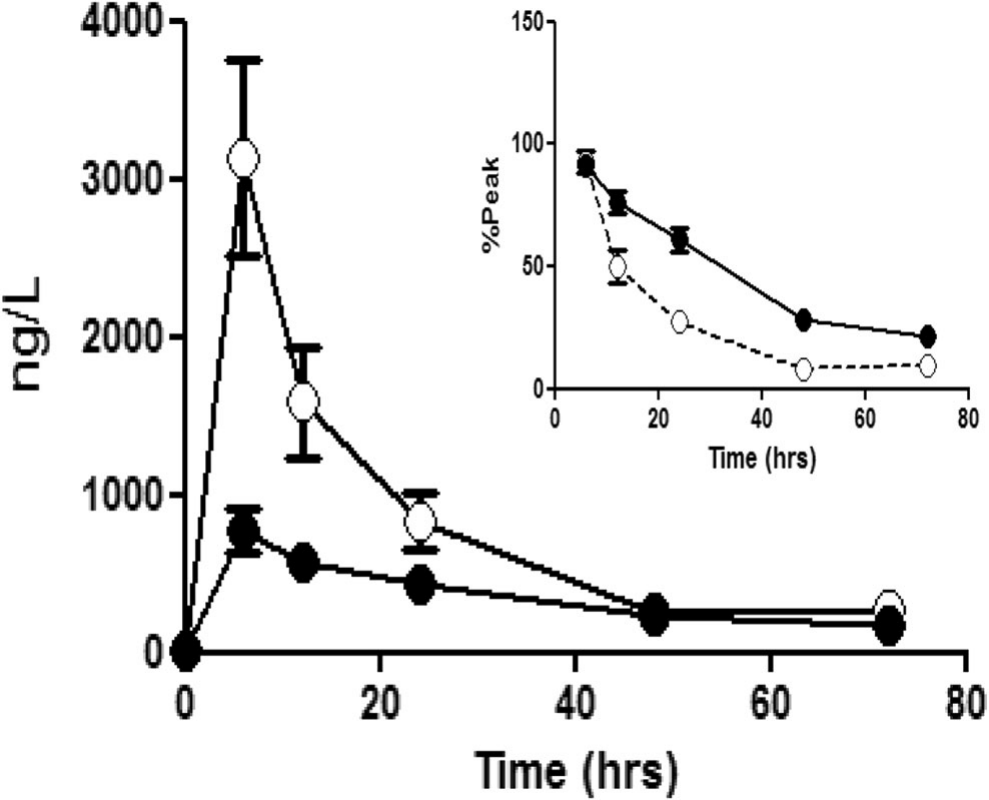
The accumulation of cMyC (open symbols) vs. cTnT (closed symbols) after myocardial injury caused by surgical revascularisation. Venous blood was collected over 3 days following CABG. Summary data of absolute quantification of cMyC vs. cTnT over time following CABG (*n* = 20). Inset figure is a zoom of the last five time points expressed as a % of peak concentration achieved in each patient. This normalisation was used to remove the visual bias caused by the greater absolute concentration of cMyC. The decay half-time for cMyC is considerably shorter than for cTnT (5.5 ± 0.8 h vs. 22 ± 5 h, *p* < 0.0001). Figure reproduced from Baker et al. [22]

Using the quantitative immunoassay described above, cMyC release kinetics were investigated in patients with ST-elevation myocardial infarction (STEMI, *n* = 20), undergoing therapeutic ablation of septal hypertrophy (TASH, *n* = 20) for hypertrophic cardiomyopathy (HCM; Fig. 4) or having coronary artery bypass surgery (CABG, *n* = 20; Fig. 5). In both models of myocardial infarction (STEMI, TASH), we detected an earlier peak of cMyC when compared to a high-sensitivity cTnT assay (STEMI, 9.3 ± 3.1 vs. 11.8 ± 3.4 h, *p* < 0.007; TASH, 9.7 ± 1.4 vs. 21.6 ± 1.4 h, *p* < 0.0001), a quicker accumulation (during first 4 h after TASH, 25.8 ± 1.9 vs. 4.0 ± 0.4 ng/L/min, *p* < 0.0001) and faster disappearance (post-CABG, decay half-time 5.5 ± 0.8 vs. 22 ± 5 h, *p* < 0.0001) [22].

These data suggest cMyC may fulfil the criteria needed to usurp troponin as described above. Figure 4 shows cMyC rises more rapidly after acute myocardial injury. Figure 5 shows cMyC falls more rapidly and this may translate into a lower background concentration in those with vascular risk factors and/or underlying chronic heart disease. Unfortunately, these data also show that the in-house assay does not have the analytic performance needed to measure cMyC in serum from healthy patients. This is required to measure the population-defined 99th centile. Hence, we commissioned a contract research company to develop an assay using the same capture/detection monoclonal antibodies, but on a high-sensitivity platform.

## Development of a High-Sensitivity cMyC Immunoassay

The new, high-sensitivity assay was developed on the Erenna platform (originally by Singulex Inc., California, USA), using the same antibody-pair (1A4, 3H8) used for the in-house assay [57]. This achieved a lower limit of detection of 0.4 ng/L and LoQ of 1.2 ng/L (20% coefficient of variation (CV), and ≤ 10% CV at 99th centile). This was used to measure cMyC in 360 stable patients without significant obstructive coronary artery disease and (hs-cTnT) < 14 ng/L. cMyC was quantifiable in 359 patients (compared to 85 and 307 patients with quantifiable hs-cTnT and hs-cTnI levels, respectively) and correlated positively with both Troponin assays (*R* = 0.56 for cTnT, *R* = 0.77 for cTnI). Further, this facilitated the calculation of the 99th centile for cMyC at 87 ng/L. The study demonstrated in stepwise multiple logistic regression analysis that age, gender, creatinine, pulmonary hypertension, as well as the use of certain medication (statins, loop diuretics, beta-blockers) all statistically predicted cMyC concentrations.

## Is There A Risk of False-Negative Results in HCM Patients?

**Fig. 6:**
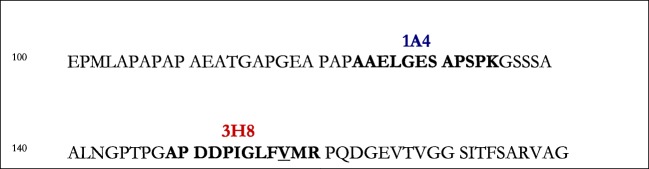
Amino acid sequence of cMyBP-C with variant MET-158 underlined; antibodies 1A4 (blue) and 3H8 (red) at binding location

As summarised above, cMyBP-C mutations causing HCM are frequent but cause either truncation mutations resulting in haploinsufficiency (thus limited expression of the protein variant) or missense mutations with a phenotypically broad range. However, most missense mutations affect the C-terminal domains of cMyBP-C, and the (purposeful) antibody alignment with the N-terminal domains C0–C1 makes it very unlikely that the newly developed assay is at risk of missing cMyBP-C elevations in a patient with HCM. The only known variant affecting a domain bound by our antibodies is MET-158, substituting valine with methionine at position 158 (target of 3H8)—felt to be a non-pathogenic polymorphism [58, 59]. The affected amino acid sequence is highlighted below (Fig. 6).

## How Sensitive Is the New High-Sensitivity cMyC Assay—Quantifying the Release of Biomarkers of Myocardial Necrosis from Cardiac Myocytes and Intact Myocardium [60]

Having commissioned a new assay for cMyC, we wanted to compare its sensitivity to the leading commercial assays for cTn. The purpose of this study was, among others, to establish the amount of cTn and cMyC release from cardiomyocytes and human cardiac tissue undergoing simulated necrosis. Serum from healthy volunteers was obtained and used as reference. Rat cardiomyocytes and human cardiac tissue were subjected to ultrasonication to simulate complete necrosis and spiked into the healthy reference serum. Samples were measured with hs-cTnI, hs-cTnT and cMyC assays (human cardiac tissue spikes only).

It was possible to detect the cTn release from the equivalent of a single cardiomyocyte with both hs-cTn assays, resulting in a slope of 19 ng L^−1^/cell (95% CI 16.8–21.2) for hs-cTnT and 18.9 ng L^−1^/cell (95% CI 14.7–23.1) for hs-cTnI. Similarly, each microgram of myocardial tissue resulted in an increase in measured hs-cTn values: 3.9 ng L^−1^/μg (95% CI 3.6–4.3) for hs-cTnT and 4.3 ng L^−1^/μg (95% CI 3.8–4.7) for hs-cTnI. cMyC generated a much greater response on the Erenna assay, with a slope coefficient of 41.0 ng L^−1^/μg (95% CI 38.0–44.0).

The results are remarkable for two reasons: First, they demonstrate the exquisite sensitivity of contemporary cardiac biomarker assays, capable of detecting release from a single cardiomyocyte, and we extrapolated that necrosis of only 40 mg of myocardium is sufficient to breach the respective 99th centiles—too little to be detected by modern cardiac tissue imaging. Second, the experiments suggest that necrosis of 3–9 mg of human myocardial tissue increases cTnT/I above the LoD as measured by high-sensitivity assays, and the corresponding value for cMyC is 0.07 mg. But how would a more sensitive assay translate into clinical practice?

## Is the Relative Abundance and Sensitivity Relevant in Clinical Practice?

We investigated the performance of the novel cMyC assay (Erenna) in 174 patients with suspected AMI, presenting very early after symptom onset [61]. All patients were part of a subgroup of individuals recruited in the HighSTEACS [62] study, presenting with chest pain of less than 3 h duration prior to first blood draw—all underwent blood draws at 0, 3 and 6-12 h (late); 26 were adjudicated with type 1 myocardial infarction.

We calculated a cMyC/hs-cTnI ratio for each of the three sampling time points. This demonstrated a positive linear correlation between the two biomarkers. However, mean and median ratios in patients with AMI were much greater at presentation than in the later timepoints (median 2.72 at 0 h, 1.83 at 3 h, 0.63 at 6–12 h), suggestive of a more dynamic rise of cMyC in the early stages of myocardial infarction than hs-cTnI. To our knowledge, no study has explored as to whether this earlier rise is due to a different release mechanism, such as cMyC release prior to cell death—similar to a myocardial stress signal—or simply a function of greater protein abundance and a very sensitive assay, allowing for earlier detection of smaller increments. Regardless, we hypothesised that this could enable more rapid and/or accurate triage. Clearly, a more in-depth evaluation of the diagnostic performance of cMyC was required in a larger study.

## Analysis of cMyC in > 1900 Patients with Suspected AMI—Direct Comparison with Cardiac Troponins

We analysed cMyC in 1954 unselected patients presenting with symptoms suggestive of AMI to emergency departments in a prospective, diagnostic multi-centre study based in Europe [27]. We focussed on studying the diagnostic properties of the presentation blood test alone and compared cMyC performance to that of hs-cTnT and hs-cTnI. The study was adjudicated using hs-cTnT and the Universal Definition of MI [63], the prognostic endpoint being long-term mortality at 3-year follow-up.

AMI was the final diagnosis in 340 patients (17%), and we observed a much greater dynamic range of cMyC in AMI versus non-AMI patients, and in comparison to both hs-cTn assays. The diagnostic performance was investigated by calculating the area under the receiver-operating characteristics curve, and cMyC matched the performance of both hs-cTn assays (cMyC AUC 0.924 vs. 0.927 hs-cTnT and 0.922 hs-cTnI). We used an internal derivation/validation split of the cohort to obtain optimal cut-offs for cMyC-guided rule-out and rule-in of AMI at presentation—10 ng/L for rule-out, 120 ng/L for rule-in. These were used to calculate a Net Reclassification Improvement, based on re-classification of patients to rule-out or rule-in categories, where cMyC was substantially more effective than either hs-cTn assay (NRI + 0.149 vs. hs-cTnT, + 0.235 vs. hs-cTnI). A remarkable signal was the higher AUC in early presenters (chest pain < 3 h) when compared to the adjudicating biomarker hs-cTnT (AUC 0.915 vs. 0.892, *p* = 0.022), also reflected in an even higher NRI in this subgroup.

This was the first study to comprehensively study cMyC performance in comparison to the best available biological signals for the diagnosis of AMI. Notably, the study was adjudicated using hs-cTnT and yet triage classification was more efficient (based on smaller observe-zone) and as accurate using cMyC. Furthermore, the patients recruited overall represent a cohort of late presenters, with a median chest pain time of 5 h prior to admission. Findings including subgroup analysis corroborate our previous observations in the HighSTEACS subgroup—a marked advantage in early presenters, with an at least as good diagnostic performance but better triage capability.

## Conclusions

Cardiac myosin-binding protein C is a novel biomarker of myocardial injury with great potential for assisting in the early rule-out of AMI—other groups [64–66] have investigated the use of cMyC in the diagnosis of myocardial infarction with confirmatory findings [20, 22], but were limited by poor assay sensitivity. Despite careful selection of monoclonal antibodies and initially promising results on our electrochemiluminescence platform, cMyC sensitivity was outperformed by the increasingly available high-sensitivity Troponin assays. Kuster et al. [64] independently reached a comparable LoD on the same device (MesoScale Discovery), making the translation of the assay onto a platform with greater sensitivity the natural next step. Given the binding sites of the two monoclonal antibodies are only affected by a single known mutation causing a non-pathogenic polymorphism of HCM, the risk of false-negative results appears diminishingly small.

Our work in migrating onto the Singulex Erenna enabled—for the first time—reliable cMyC quantification in stable outpatients. As demonstrated [57], this assay enabled two leaps in the translational phase: (1) quantify the cMyC level in all but one of 360 individuals without acute cardiovascular disease, thus allowing (2) the derivation of a 99th centile (87 ng/L, as published [57]). The assay, performed by a contract research organisation, achieved a LoD 200 times lower than our in-house assay and laid the foundation for clinical studies as described.

Favourable release kinetics and a higher sensitivity than hs-cTn assays are likely responsible for the better performance in patients presenting early after chest pain onset [57, 60, 61]. The greater analytic bandwidth of the assay could, in turn, be responsible for a better calibration against acute myocardial injury versus the chronic release of myocardial necrosis markers often observed in clinical practice [27]. This would explain the net reclassification benefit observed in the largest cohort study testing cMyC to date—both in all-comers and early presenters [27]. As demonstrated in a single-centre prospective cohort study investigating the use of hs-cTnT in the emergency department at our institution [67], 52% of patients are assigned to an ‘observe’ zone after the first blood draw (~ 4000 patients annually; triage modelled on the 2015 ESC NTEMI guidelines [13]). These patients—quasi-automatically—require repeat blood testing and therefore ongoing observation until a level of diagnostic certainty can be reached. Any admission avoided, employing more dynamic but equally specific cardiac necrosis markers, should be in the best interest of healthcare providers and patients alike. Extrapolating from findings to date, the gains might not be marginal!
